# Development of a unique anti-AML immune therapy consisting of cord blood HSCT and cord blood stem cell-derived dendritic cell (CB-DC) vaccination

**DOI:** 10.1186/2051-1426-1-S1-P206

**Published:** 2013-11-07

**Authors:** Colin de Haar, Maud Plantinga, Nina Blokland, Lotte Spel, Marianne Boes, Stefan Nierkens, Jaap Jan Boelens

**Affiliations:** 1 U-DANCE, Tumor Immunology Section, Laboratory of Translational Immunology, UMC Utrecht, Utrecht, the Netherlands

## Background

Development of novel (immune) therapies is of utmost importance to improve survival in relapsed pediatric-AML (acute myeloid leukemia). We aim to develop a powerful and safe therapy consisting of 2 synergistic components: Cord Blood (CB) HSCT and vaccination with CB-derived Wilms Tumor-1 (WT1) mRNA-electroporated dendritic cells (DCs).

## Materials & methods

After isolation, the CD34+ CB stem cells were cultured using a two-step protocol. First, they were expanded using a combination of (growth) factors (Flt3L, SCF, IL-3 and IL-6). Next, the cells were differentiated towards DCs for one week using medium containing Flt3L, SCF, GM-CSF, IL-4 and human serum followed by a CYTOMIX (IL-1β, IL-6, TNF-α and PGE2)-induced maturation for the last 24 hours. Finally, the CB-DC culture was electroporated with WT1-mRNA and their phenotype (cell surface markers) and function (migration and antigen presentation) were assessed.

## Results

Using the two-step protocol a total cell expansion of 300-500 fold was achieved. Based on surface marker expression, at least 5 different DC subsets could be distinguished in our CB-DC cultures. Since no differences in antigen presentation capacity between the DC subsets were detected, the whole CB-DC culture was used in all phenotypic and functional assays. The maturation using CYTOMIX induced upregulation of costimulatory molecules and CCR7. These cells were also functional, showing enhanced CCR7-dependent migration towards CCL21 in a trans-well migration assay. Finally, the stimulation of WT1-specific T cells by the CB-DCs, matured using CYTOMIX and electroporated with WT1 mRNA, confirmed presentation of WT1 antigens.

## Conclusion

We have developed and tested an in vitro system for culturing large amounts of DCs from the CD34+ CB stem cells. Both the phenotypic and functional data support the use of the whole CB-DC culture as vaccine. The next step will be to translate the preclinical protocol to GMP production of a clinical grade vaccine.

